# Cost-effectiveness analyses of self-harm strategies aimed at reducing the mortality of pesticide self-poisonings in Sri Lanka: a study protocol

**DOI:** 10.1136/bmjopen-2014-007333

**Published:** 2015-02-27

**Authors:** Lizell Bustamante Madsen, Michael Eddleston, Kristian Schultz Hansen, Melissa Pearson, Suneth Agampodi, Shaluka Jayamanne, Flemming Konradsen

**Affiliations:** 1 Section of Global Health, Department of Public Health, Faculty of Health and Medical Sciences, University of Copenhagen, Copenhagen, Denmark; 2 Pharmacology, Toxicology & Therapeutics, University/BHF Centre for Cardiovascular Science, University of Edinburgh, Edinburgh, UK; 3 South Asian Clinical Toxicology Research Collaboration, Faculty of Medicine, University of Peradeniya, Peradeniya, Sri Lanka; 4 Department of Global Health and Development, London School of Hygiene and Tropical Medicine, London, UK; 5 Department of Community Medicine, Faculty of Medicine and Allied Sciences, Rajarata University, Saliyapura, Sri Lanka; 6 Department of Medicine, Faculty of Medicine, University of Kelaniya, Ragama, Sri Lanka

**Keywords:** HEALTH ECONOMICS, PUBLIC HEALTH

## Abstract

**Introduction:**

An estimated 803 900 people worldwide died as a result of self-harm in 2012. The deliberate ingestion of pesticides has been identified as the method most frequently used to commit fatal self-harm globally. In Sri Lanka, it is estimated that up to 60% of all suicides are committed using this method. The aim of the present study is to assess the cost-effectiveness of an ongoing safe storage intervention currently taking place in a rural Sri Lankan district and to model the cost-effectiveness of implementing the safe storage intervention as well as four potential interventions (legislative, medical management, follow-up contact and mobile phone contact) on a national level.

**Methods and analysis:**

Study design for all the strategies is a cost-effectiveness analysis. A governmental perspective is adopted. The time horizon for tracking the associated costs and health outcomes of the safe storage intervention on district level runs over 3 years. The time horizon is extended to 5 years when modelling a full national roll-out of the respective interventions. The discounting of costs and health outcomes are undertaken at the recommended real rate of 3%. Threshold analyses of the modelled strategies are employed to assess the strategies potential for cost-effectiveness, running scenarios with health outcome improvements ranging from 1% to 100%. Sensitivity analyses are also performed. The main outcome measures of the safe storage intervention are incremental cost-effectiveness ratios.

**Ethics and dissemination:**

Ethical approval was granted for the safe storage project from the University of Peradeniya, Sri Lanka, in March of 2008. An amendment for the present study was granted from Rajarata University of Sri Lanka in November of 2013. Findings will be disseminated to public and private stakeholders in local and national government in Sri Lanka as well as the wider academic audience through peer-reviewed publications and international conferences.

**Trial registration number:**

The safe storage cluster trial is registered with the Clinical Trials, ref: NCT1146496 (http://clinicaltrialsfeeds.org/clinical-trials/show/NCT1146496).

**Strengths and limitations of this study**

- The study will generate new knowledge to an under-researched topic, that is, the cost-effectiveness of interventions aimed at reducing the mortality of pesticide self-poisoning.
- The strength of the study lies in the randomised design of the safe storage trial and the large sample size which allows sufficient power to assess the intervention's effect on self-harm.
- The chosen type of analysis and outcome measure narrows the field of comparisons with other types of interventions.
- Analyses of potential preventive strategies aimed at reducing the mortality of pesticide self-poisoning are reduced to modelling and threshold analyses due to the limited data available.

## Introduction

The WHO has estimated that 803 900 people worldwide died as a result of self-harm in 2012.^1^ In the 2010 Global Burden of Disease Study, self-harm was identified as the second most important type of injury reported and it was among the 25 leading causes of disease burden.^2^

The WHO has recognised deliberate ingestion of pesticides as the most frequently used method of self-harm globally.^3^ In a systematic review from 2007, it was estimated that pesticide self-poisoning was responsible for as much as one-third of all suicides globally.^4^ In China, it is estimated that as much as 58% of all suicides are due to deliberate pesticide ingestion,^5^ while Patel *et al*^6^ recently found that the method was employed in approximately half of all suicides in India.

In 1995, Sri Lanka had one of the highest suicide rates in the world with approximately 47 suicides per 100 000 population.^7^ The country has had great success in reducing the suicide incidence rates through bans of the most toxic pesticides, and in 2005, suicide rates had fallen to 24/100 000.^7^ A retrospective study of in-patient records in southern rural Sri Lanka from 1990 to 2002 found that 61% of all acute poisoning cases admitted to hospital were due to pesticide ingestion.^8^ Police records for 2011 showed that 46% of all suicides registered were due to the ingestion of pesticides and insecticides.^9^ Suicide by deliberate ingestion of pesticides therefore continues to be a major public health issue in Sri Lanka as an important cause of premature mortality.

It is hypothesised that pesticide ingestion contributes so heavily to the patterns of suicide in low-income countries because of the easy accessibility to pesticides in farming households and in nearby shops as well as the high overall case fatality, which ranges from less than 5% to 70%, depending on the pesticides used.^4^ ^10–12^ Yang *et al*^5^ found that 61% of all fatal pesticide self-poisoning cases in China were due to unsuccessful medical resuscitation.

There is an urgent need for strategies aimed at preventing deaths from pesticide self-poisoning in Sri Lanka, as well as the rest of the region.^4^ ^13^ A self-harm prevention strategy that has been suggested in the literature is the restriction of access to means.^14^ ^15^ With regard to self-harm using pesticides, it has been recommended by the WHO, and in the literature, to restrict the availability of highly toxic pesticides, for example, by banning the production, import and use of a pesticide, or to restrict access to pesticides by keeping them in locked containers, and reduce the use of pesticides in agricultural practices.^12^ ^13^ ^16–18^ A suggested treatment strategy involves improvement of the medical management of the poisoning cases by ensuring that hospitals have adequate supplies of antidotes, respirators and trained personnel.^12^ ^17^ ^19^ Finally, previous attempts at self-harm is a risk factor for repetition, and findings suggest that postinterventions focusing on follow-up contract with people who have previously attempted self-harm could reduce suicide rates.^14^ ^20–22^

For decision-makers to determine which health-related interventions to implement in their country, several factors must be taken into consideration, including public health and economic considerations. The direct and indirect economic costs of self-harm and suicide include emergency services and intensive hospital care as well as costs of lost productivity. A study from Sri Lanka estimated the total direct cost to the Ministry of Health of treating self-poisoned patients to be US$1 072 571.^23^ Studies from Scotland and Ireland estimated the cost per suicide to society to be US$3 067 463 and US$2 066 886, respectively (inflationary adjustments have been made to bring all costs to year 2013).^24^ ^25^ The pain and grief that family members and friends suffer can also be immense and affect their ability to function in society.^12^ The extensive costs of pesticide self-poisonings to families, society and the general economy therefore suggest a strong economic incentive for investment in self-harm interventions.

Presently, very little evidence exists on the cost-effectiveness of interventions aimed at reducing the mortality from pesticide self-poisoning which could be used to prioritise their introduction. The overall aim of the study is to address this evidence gap by (1) assessing the cost-effectiveness of an ongoing safe storage intervention currently taking place in rural Sri Lanka; (2) assessing the cost-effectiveness of implementing the safe storage intervention on a national level and (3) modelling the cost-effectiveness of implementing four potential interventions (legislative, medical management, follow-up contact (physical contact through a volunteer organisation and mobile phone contact)) on a national level. In the following, the outline of the economic evaluation of all interventions is presented.

## Methods and analysis

### Overview of the strategies

#### Safe storage intervention

The focus of the research is an ongoing community-based, cluster randomised controlled trial (RCT) which is presently being conducted in the Anuradhapura district of Sri Lanka's North Central Province. The aim of the RCT is to evaluate the effectiveness of storing pesticides in specially designed lockable containers in reducing the mortality of pesticide self-poisoning as well as the overall incidence of self-harm, and mortality following self-harm.^26^ Access to pesticides is restricted by storing them in lockable containers which are then partially buried underground outside the house. The full details on the design and methods employed in the RCT are provided elsewhere (see ref. 26 for details). The trial is registered on Clinical Trials, ref: NCT1146496 (http://clinicaltrialsfeeds.org/clinical-trials/show/NCT1146496).

##### Study sample and sample size calculation

**Figure 1:**
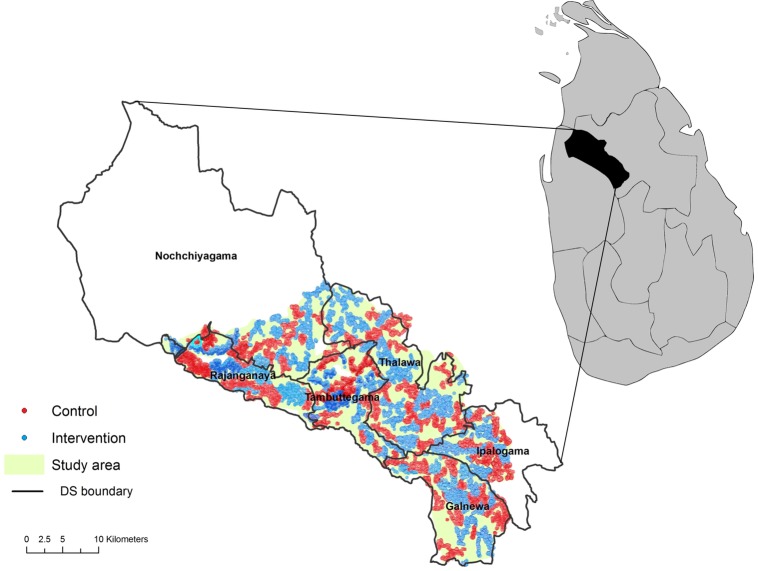
Distribution of study areas in the Anuradhapura district of Sri Lanka (map of Sri Lanka adapted from ref. 26; DS, divisional secretariat).

The trial has recruited 53 471 households from a total of 180 clusters since 31 December 2010 from the Mahaweli H region in the North Central Province in Sri Lanka, including the divisional secretariats of Tambuttegama, Talawa, Galnewa, Rajanganaya, Nochchiyagama and Ipalogama (see figure 1). The randomisation unit was the clusters, primarily villages, which were allocated to either the intervention or control group.^26^

All households in the study area were approached, briefly introduced to the trial and then they were invited to give verbal consent to participation. Inclusion criteria were households with a resident farmer, or reported use of pesticides or storage of these. Exclusion criteria included villages that had been recruited to previous pilot studies of pesticide safe storage, and households without an adult available to provide consent.^26^

Under the assumptions that 20% of individuals in the intervention arm lived in households not using a lockable container, and 5% of individuals in the control arm lived in a household using one, it was estimated that 223 925 person years of follow-up were required in each arm to detect a 30% reduction in pesticide self-poisoning with 80% power at the 5% significance level.

#### Legislation

In 1995, the Sri Lankan government implemented a national policy banning all pesticide formulations of WHO hazard class Ia/Ib.^27^ In the legislative strategy, it is assumed that all pesticide formulation(s) of WHO hazard class I and II (moderately hazardous) are banned from production, import and use, and another product is recommend by the Department of Agriculture to substitute these products. The costs of implementing and enforcing a policy banning all class I and II pesticide formulations used for the most commonly grown crops and vegetable products are assessed.

#### Medical management

In the treatment strategy, it is assumed that case management of pesticide self-poisoning can be improved by ensuring that peripheral hospital facilities have adequate supplies of drugs, including antidotes, ventilators, laboratory facilities and other necessary equipment to treat and transfer patients. In the analyses, it is determined what such essential packages should entail and how many of these are needed in the hospital facilities at each level. The strategy also includes targeted training of health personnel in pesticide poisoning case management, such as following poisoning guidelines.

#### Follow-up contact postintervention

In the analysis of the follow-up contact strategy, it is assumed that repeated attempts of pesticide self-poisoning among previous self-harmers can be prevented by offering them increased social and emotional support, that is, offering assistance and companionship as well as counselling on alcohol and drugs, through a volunteer organisation such as the local branch of Befriender's Worldwide, Sumithrayo.

#### Mobile phone-based postintervention

In the mobile phoned-based intervention, it is likewise assumed that using mobile phones and daily texts to follow-up on patients who have previously self-harmed can prevent repeated episodes of self-harm.

The approaches to prevent mortality from pesticide self-poisoning are summarised in table 1.

**Table 1:** Description of interventions

Preventive approach to reduce mortality from pesticide self-poisoning	Intervention	Description
Primary prevention	Safe storage	Provide farmers with lockable devices to safely store pesticides outside of households
	Legislation	Ban all pesticide formulation(s) of WHO hazard class I and II (moderately hazardous) from production, import and use
Tertiary prevention	Medical management	Improve medical management of pesticide self-poisoning cases by ensuring that hospitals have the essential equipment and drugs and that health personnel are properly trained
Postintervention	Follow-up	
	Volunteer organisation	Prevent repetitious attempts of self-harm among previous self-harmers by offering them physical, social and emotional support from volunteers
	Mobile phone	Prevent repetitious attempts of self-harm among previous self-harmers by offering them social contact through phone conversations and daily text messages

### Economic evaluation of self-harm interventions

#### Outline of the economic evaluation

The economic framework for all the strategies is a cost-effectiveness analysis (CEA). A governmental perspective is adopted for the economic evaluations, so only cost and outcomes that impact on government as a third party funder are included while all costs and outcomes for patients are excluded. The comparator in all the analyses is status quo, that is, it is assumed that no current practice, beyond present level of medical case management, is in place, and all costs and health outcomes associated with the implementation of the respective strategies are compared with the costs and health outcomes in the absence of all interventions.

In the economic evaluation of the safe storage intervention, a 3-year CEA is conducted corresponding to the follow-up period of the intervention. In the modelling of a full national roll-out of the respective interventions, the time horizon for tracking the associated costs and health outcomes is extended to a 5-year period.

All costs are expressed in US$ and measured in real prices for the reference year (2010) using the gross domestic product deflator. If this is not available, the consumer price index will be used. The discounting of costs and health outcomes is undertaken at the recommended real rate of 3% to take into account the timing of costs and health outcomes of the intervention that does not occur in the present.^28–30^

All participants recruited in the RCT are included in the economic evaluation of the safe storage intervention. When determining the potential cost-effectiveness of the respective strategies on a national scale, data are extrapolated to the actual Sri Lankan population.

#### Estimating cost

In accordance with the study perspective, all direct costs related to the implementation of the strategies and to the healthcare system are included in the analysis. Direct costs are divided into non-health and healthcare costs. Non-healthcare costs relate to the costs associated with the implementation of the interventions, while healthcare costs are the explicit expenses to the health sector of treating fatal and non-fatal cases of pesticide self-poisonings. The medical treatment interventions technically fall under the category of healthcare costs but are presented under direct non-health costs along with the other interventions.

All cost and cost offsets relevant to the study perspective are identified, quantified and ascribed as a unit cost to estimate the economic burden of pesticide self-poisoning and the cost of implementing the strategies. The cost components for all interventions are divided into the following categories: capital costs, personnel costs, overhead, consumables and transportation costs.

Unit costs and prices will be obtained from official statistics, providers, health facilities, the Medical Supply Division of the Ministry of Health and the Provincial Department of Health. If reliable data are not available, case regional estimates on unit costs will be obtained from the WHO-CHOICE database and through personal communication with researchers in the field. The wage rate per capita per day is derived from national wage rates.

#### Direct non-health costs

##### Safe storage intervention

Data from the safe storage project are used as basis for costing the intervention. All costs associated with the implementation, delivery and follow-up on use of the safe storage intervention are included. Research costs associated with the intervention are excluded from the analyses.

Primary data on resource utilisation directly associated with the implementation of the intervention, for example, container manufacture, delivery, personnel time dedicated to distributing containers and training farmers how to use them, are available in the safe storage trial databases. Additional items and resource utilisation associated with the intervention will be identified and assessed by reviewing the safe storage protocols and through quantitative interviews with key informants from the project.

The cost of the intervention is calculated by multiplying the resource utilisation of the identified input parameters with the relevant unit costs or prices. Travel cost is estimated on the basis of average distance to the villages, frequency of visits and fuel prices. Annual recurrent costs of spare parts and maintenance of capital costs and the wastage rate of safe containers are assessed and included in the analysis.

Table 2 presents an overview of identified cost components and items that are included in the analyses.

**Table 2:** Costing the safe storage intervention

Cost category	Type of cost	Cost components	Cost items	Cost unit
Direct	Non-healthcare	Capital costs	Vehicles IT equipment Storage containers, including padlock and three keys	Numbers of units purchased, unit price
		Personnel	Distribution team Community mobilisation team Drivers Contract personnel Administrative personnel	Salaries, wage rates, man hours
		Overheads	Rent: Storage facilities Office facilities Telephone and internet Gas, electricity and water Maintenance cost of equipment	Resource utilisation, unit price
		Consumables	Posters Information and invitation letters	Resource utilisation, unit price
		Transportation	Renting vehicles Fuel	Resource utilisation, unit price

##### Legislative strategy

It is anticipated that the input parameters for this strategy will primarily include resource utilisation in the design phase, that is, research, information gathering and consensus building, and the implementation phase. It is furthermore assumed that the Department of Agriculture is financing the majority of the expenses of implementing this strategy. Relevant cost items pertain to the running of the Pesticide Technical Advisory Committee, creating guidelines for use of pesticides to replace the class II pesticides, implementation, campaigning and the enforcement of the new policy. The specific cost items and resource utilisation associated with the strategy are identified and assessed using the literature and through quantitative interviews with key informants from the Office of the Registrar of Pesticides, the Department of Agriculture and the Institute of Policy Studies. Additional costs of the legislation beyond the design phase, implementation and enforcement of the policy are excluded. It is assumed that the pest control effectiveness of the substitute product equals that of the banned products, and that the substitution does not have any negative impact on yields.

The cost of the intervention will be calculated by multiplying resource use of the identified parameters with the unit cost or prices.

##### Medical management

Cost items and resource use directly associated with the medical management strategy, such as, additional health personnel, ventilators and ambulances needed and time spent training new personnel, are identified and assessed using quantitative interviews with key informants, for example, health and administrative personnel at hospitals. The interviews focus on resource utilisation of equipment and medical supplies as well as personnel time (see table 3 for preliminary input parameters). Additional equipment needs required beyond ventilators and laboratory equipment to establish intensive care unit level support are likewise assessed.

**Table 3:** Costing the medical management strategy

Cost category	Type of cost	Cost components	Cost items	Cost unit
Direct	Healthcare	Capital costs	Ventilators Laboratory facilities Ambulances	Numbers of units purchased, unit price
		Personnel	Additional health personnel needed in facilities at each level Additional drivers Administrative personnel	Salaries, wage rates, man hours
		Overheads	Maintenance cost of equipment	Resource utilisation, unit price
		Consumables	Drugs Materials and supplies Laboratory tests and examinations	
		Transportation	Fuel	Resource utilisation, unit price

The cost of the intervention is calculated by multiplying the resource utilisation of the identified input parameters with the relevant unit cost or price. Transfer cost is estimated on the basis of average distance travelled, frequency of transfers and fuel prices. Annual recurrent costs of spare parts and maintenance of capital costs are estimated as 5% of the total capital cost. Wastage rates of supplies are assessed and included in the analysis.

##### Follow-up contact

Cost items and resource utilisation associated with the follow-up intervention are identified and assessed using quantitative interviews with key informants from the Sri Lankan branch of Befriender's Worldwide, Sumithrayo. The preliminary input parameters included in the analysis are presented in table 4.

**Table 4:** Costing the follow-up intervention

Cost category	Type of cost	Cost components	Cost items	Cost unit
Direct	Non-healthcare	Capital costs	Vehicles IT equipment	Numbers of units purchased, unit price
		Personnel	Administrative personnel Volunteers supervision	Salaries, wage rates, man hours
		Overheads	Telephone and internet Maintenance costs of	Resource utilisation, unit price
			Equipment	
		Consumables	Training material Office supplies	Resource utilisation, unit price
		Transportation	Fuel	Resource utilisation, unit price

The cost of the intervention is calculated by multiplying resource use of identified parameters with the unit cost or prices. Transportation costs are assessed on the basis of fuel costs, average distance travelled and frequency of follow-up visit. Annual recurrent costs of spare parts and maintenance are calculated as described in previous sections.

##### Mobile phone contact

Table 5 presents an overview of input parameters included in the mobile phone intervention. Additional cost items and resource utilisation associated with the intervention will be identified and assessed using existing databases and through quantitative interviews with researchers within the field.

**Table 5:** Costing the mobile phone intervention

Cost category	Type of cost	Cost components	Cost items	Cost unit
Direct	Non-healthcare	Capital costs	IT equipment Mobile phones	Numbers of units purchased, unit price
		Personnel	Administrative personnel Health personnel	Salaries, wage rates, man hours
		Overheads	Telephone and internet	Resource utilisation, unit price
		Consumables	Office supplies Training materials	Resource utilisation, unit price

The cost of the intervention is calculated by multiplying resource use of identified parameters with the unit cost or prices.

#### Direct healthcare costs

Costs to the health sector can be divided into hospital treatment (inpatient and outpatient), outpatient healthcare, and transportation to and from hospital.^31^

The cost of direct healthcare is calculated by identifying resource use of relevant healthcare input parameters, for example, the number of hospital inpatient and outpatient admissions, as well as the average length-of-stay in hospital; use of drugs and laboratory tests; number of examinations; number of transfers and distance travelled, and multiplying them with the relevant healthcare unit prices. The average length-of-stay at hospital and average cost per day of pesticide self-poisoned patient is estimated using hospital data. The allocation of joint costs for wards, for example, administration/support service cost, rental cost of premises and overheads, is based on number of pesticide self-poisoning patients.

In a previous study from Sri Lanka, the direct costs to the Sri Lankan Ministry of Health of treating self-poisoned patients in a single district in 2005/2006 were estimated.^23^ This database is consulted to identify cost items and resource utilisation. Additional data and relevant input parameters are obtained from hospital data systems and facility registries and through quantitative interviews with relevant hospital personnel.

Table 6 presents a brief overview of the cost components and items included in the costing of the direct healthcare costs of treating pesticide self-poisoning cases.

**Table 6:** Healthcare costs

Cost category	Type of cost	Cost components	Cost item	Cost unit
Direct	Healthcare	Capital costs	Ventilators Laboratory facilities Ambulances Buildings	Resource utilisation, unit price
		Personnel	Health personnel Non-medical personnel, including drivers and administrative personnel	Salaries, wage rates, man hours
		Overheads	Maintenance cost of equipment Electricity, gas and water	Resource utilisation, unit price
		Consumables	Drugs Materials and supplies Laboratory tests and examinations	Resource utilisation, unit price
		Transport	Fuel	Resource utilisation, unit price

#### Data collection of fatal and non-fatal pesticide self-poisoning cases

Data on fatal and non-fatal cases of pesticide self-poisoning are currently being collected by the safe storage team from death registries, mortuary and hospital records, hospital admission and emergency department's registries as well as police records.

#### Estimating health outcomes

The primary analysis of the safe storage intervention follows the intention-to-treat principle, comparing the observed incidence of pesticide self-poisoning between individuals in villages allocated to the intervention, and individuals in villages allocated to the control arm. As described in Pearson *et al*,^26^ a Poisson regression model is employed in the analysis, with the SEs inflated to accommodate the clustered design. The analysis is adjusted for minimisation variables used in the random allocation and for seasonal variation in the incidence of pesticide self-poisoning. This same approach is used for the secondary outcome measures.^26^

In the safe storage intervention, the primary outcome is the incidence of pesticide self-poisoning, both fatal and non-fatal, among villagers aged 14 years or older, while secondary outcomes include incidence of pesticide poisoning in general, self-harm, self-poisoning (all substances) and pesticide poisoning in children (younger than 14 years).^26^ Effectiveness data for the safe storage project are used to estimate the denominator of the economic evaluation ratio, that is, number of fatal pesticide self-poisoning cases prevented, number of pesticide self-poisoning deaths prevented and life-years saved due to the intervention.

The analyses of the potential strategies are likewise carried out according to the intention-to-treat principle. However, the possibility of conducting a full economic evaluation of the legislative, medical management or follow-up is severely restrained by the limited data available on the effectiveness of such strategies. Threshold analyses are employed to model the potential for cost-effectiveness by ranging the effectiveness of each analysis in reducing death by pesticide self-poisoning from 1% to 100%.

#### Assessing cost-effectiveness

Incremental cost-effectiveness ratios (ICERs) are calculated to estimate the cost-effectiveness of the safe storage intervention and the potential for cost-effectiveness for the alternative strategies compared with status quo. ICERs are calculated as the ratio of the incremental difference in total costs between the intervention group and the control group (ΔC) that is divided by the difference in effects (ΔE):
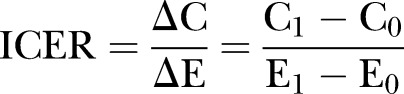


The primary outcome of the cost-effectiveness analyses is cost per life year saved (LYS)

#### Uncertainty and sensitivity analysis

One-way sensitivity analyses are undertaken to assess how parameter uncertainties impact on the cost-effectiveness of the strategies, thereby identifying the factors affecting the total cost of implementing the strategy. This involves varying individual input parameters’ value across a range of 10–100% and assessing how this changes the analysis’ results.^28^ Multivariate sensitivity analyses are also performed to assess how simultaneous changes of several variables affect the cost-effectiveness ratio. Probabilistic uncertainty analyses are performed to explore the impact of variability in (1) input parameters that can be measured, and (2) input parameters for which there is an underlying probability distribution.

To investigate discounting scenarios other than the 3% rate, sensitivity analyses are run with 0% and 6% discount rates.^30^

## Ethics and dissemination

An amendment for the present study was granted from Rajarata University of Sri Lanka in November of 2013.

The results of the study will be disseminated to stakeholders in local and national government in Sri Lanka as well as the wider academic audience and medical communities through peer-reviewed publications and international conferences.

## Discussion

The present paper outlines the economic evaluation of a pesticide safe storage intervention and four potential strategies aimed at reducing mortality of pesticide self-poisoning in Sri Lanka. The study aims to study the cost-effectiveness of implementing such strategies to aid decision-makers and policy-makers in making well-informed policy decisions to prevent death from pesticide self-poisoning.

To the best of our knowledge, this will be among the first economic evaluations undertaken for strategies aimed at reducing the mortality of pesticide self-poisoning. As such, this study will contribute positively to the evidence base of an erstwhile under-researched topic.

The strength of the study lies in its large sample size and the randomised design of the safe storage trial. Data on pesticide self-poisoning cases are currently being collected by the safe storage team, thereby ensuring that primary data sources are employed in the study.

There are several limitations of this study. The chosen analysis and outcome measure, cost per LYS only focus on mortality, excluding morbidity or quality of life aspects. This focus limits the possibility of comparing the outcome of suicide prevention, that is, a strategy that targets a fatal condition, with interventions targeting chronic conditions such as diabetes. The choice of a governmental perspective will likely affect the cost-effective ratio of the interventions as it excludes the cost of lost productivity. According to the WHO, suicide is the second leading cause of mortality among 10–24-year olds, which means that society experience a huge productivity loss due to their premature deaths.^32^ A study by Choi *et al*^33^ found that the total societal cost of occupational and deliberate pesticide poisoning in South Korea was US$150 million in 2009, and that 90.6% of these costs were attributed to lost productivity.

It is not possible to include all the ‘spill-over effects’, that is, unexpected effects, which will occur as a result of the interventions and spill over to other sectors and society. It is furthermore impossible to include every single cost and health effects of the strategy, and in the analyses, some items will have to be included or excluded for practical reasons. Further limitations involve the exclusion of the trade-off between agricultural benefits of pesticides and lives saved in the legislative strategy; all-of-life effects, that is, unrelated ongoing healthcare costs of people who are alive because of the intervention; all costs to the private sector due to changes in pesticide regulation; other non-health sector impacts; and the general cost of educating and training health professionals in Sri Lanka.

Finally, the analyses of the potential strategies are reduced to modelling and threshold analyses due to the severely limited data available on the effectiveness of such strategies. However, the information that these analyses will yield can be used by decision-makers to evaluate the risk of implementing suicide interventions by giving them the tools to determine whether or not the potential gains of the interventions justify the investments.

There is a great need for more economic evaluations of suicide prevention strategies globally, but especially for low-income countries in the South East Asian region, to guide decision-makers and policy-makers to implement such strategies as well as prioritising which strategies should be allocated scarce resources.
